# Risk factors of postoperative complications after radical cystectomy with continent or conduit urinary diversion in Armenia

**DOI:** 10.1186/s40064-016-1757-9

**Published:** 2016-02-20

**Authors:** Arman Tsaturyan, Varduhi Petrosyan, Byron Crape, Yeva Sahakyan, Lusine Abrahamyan

**Affiliations:** School of Public Health, American University of Armenia, 40 Marshal Baghramyan Ave., 0019 Yerevan, Armenia; Armenia Republican Medical Center, Yerevan, Armenia; Nazarbayev University School of Medicine, Astana, Kazakhstan; Toronto Health Economics and Technology Assessment (THETA) Collaborative, University of Toronto, Toronto, Canada

**Keywords:** Radical cystectomy, Bladder cancer, Complications, Risk factors, Mortality

## Abstract

To estimate the surgical volume and the incidence of in-hospital complications of RC in Armenia from 2005 to 2012, and to investigate potential risk factors of complications. The study utilized a retrospective chart review in a cohort of patients who had RC followed by either continent or conduit urinary diversion in all hospitals of Armenia from 2005 to 2012. A detailed chart review was conducted abstracting information on baseline demographic and clinical characteristics, surgical procedural details, postoperative management and in-hospital complications. Multivariable logistic regression analysis was applied to estimate the independent risk factors for developing ‘any postoperative complication’. The total study sample included 273 patients (mean age = 58.5 years, 93.4 % men). Overall, 28.9 % (n = 79) of patients had at least one in-hospital complication. The hospital mortality rate was 4.8 % (n = 13). The most frequent types of complications were wound-related (10.3 %), gastrointestinal (9.2 %) and infectious (7.0 %). The ischemic heart disease (OR = 3.3, 95 % CI 1.5–7.4), perioperative transfusion (OR = 2.0, 1.1–3.6), glucose level [OR = 0.71 (0.63–0.95)], and hospital type (OR = 2.3, 95 % CI 1.1–4.7) were independent predictors of postoperative complications. The rate of RC complications in Armenia was similar to those observed in other countries. Future prospective studies should evaluate the effect of RC complications on long-term outcomes and costs in Armenia. Policy recommendations should address the issues regarding surgeon training and hospital volume to decrease the risk of RC complications.

**Electronic supplementary material:**

The online version of this article (doi:10.1186/s40064-016-1757-9) contains supplementary material, which is available to authorized users.

## Background

Urinary bladder cancer is the most often occurring cancer in the urinary system ranking ninth in overall cancer incidence worldwide (Parkin 2008; Janković and Radosavljević 2007). The reported incidence of bladder cancer varies widely between countries and reporting sources, mostly due to differences in disease classification (Parkin 2008; Crow and Ritchie 2003).

According to 2011 national statistics, in Armenia the incidence rate of bladder cancer was 23.9 per 100,000 population among men and 1.8 among women (National Information Analytic Centre 2012). According to worldwide incidence statistics, in 2012 Armenia was ranked 12th in age-standardized incidence of bladder cancer when considering both sexes (12,3/100,000) and 4th when considering males (27.3/100,000) (Ferlay et al. 2014). Among other important risk factors (e.g., age, occupation, genetic predisposition, schistosomiasis), smoking is the most important contributing factor for developing bladder carcinoma, with an established population attributable risk of 50 % in men and 52 % in women (Parkin 2008; Janković and Radosavljević 2007; Freedman et al. 2011). Armenia has the third highest smoking prevalence rate in the world at 27 % prevalence in the total population, with 52 % in males and 1.7 % in females in 2012 (Ng et al. 2014).

Bladder cancer mortality rates vary depending on its stage at the time of diagnosis (i.e., localized, regional or distant) with an overall 5-year disease-specific survival approaching 81 % (Abdollah et al. 2013). At the time of diagnosis, 30 % of bladder cancers are already muscle invasive and require a combined treatment of surgery with chemotherapy and/or radiation therapy (Witjes et al. 2014). The surgery includes radical cystectomy (RC) with regional bilateral lymph node dissection, and carries high risk of early and late complications due to simultaneous involvement of urinary tract, intestines, lymph nodes and the type of urinary diversion (Witjes et al. 2014). In the published literature, the proportion of patients experiencing at least one early in-hospital complication after RC varies from 26 to 67 %, with the in-hospital mortality varying from 0.6 to 2.6 % (Hollenbeck et al. 2005; Konety et al. 2006; Novotny et al. 2011; Shabsigh et al. 2009). The reported proportion of patients with complications within 90 days of the index hospitalization varies from 58 to 64 %, and mortality from 2.3 to 2.7 % (Shabsigh et al. 2009; Hautmann et al. 2010). These postoperative complications are associated with longer hospital length of stay (LOS), increased costs of care and mortality (Novotny et al. 2011; Konety and Allareddy 2007). Early perioperative outcomes vary by the type of urinary diversion with diversion-unrelated complications being high in the ureterocutaneostomy patients and diversion-related complications in continent and conduit diversion patients (Pycha et al. 2008).

Unlike many other countries, there are no comprehensive population-based, administrative or clinical databases in Armenia to capture patient care processes. So, it is unclear at what stages patients are referred to surgery, what are their outcomes, and if there have been any changes over time. The aim of the present study was to estimate the surgical volume of RC conducted for bladder cancer in Armenia from 2005 to 2012, assess the incidence of early postoperative (in-hospital) complications, and investigate potential risk factors for these complications.

## Results

We identified eight hospitals that performed RC in Armenia, all of which were located in Yerevan (i.e., none of the hospitals outside the capital had capacity to perform RC). All but one hospital performing 1–2 surgeries per year agreed to participate in the study. The medical records of all patients who had RC followed by either continent or conduit urinary diversion between 2005 and 2012 in these seven hospitals have been reviewed. After excluding 50 patients with bilateral uretrocutaneostomy, the total sample included 273 patients.

Table 1 presents patient baseline and operative characteristics. The average age of the patients was 58.5 years [standard deviation (SD) = 8.9] and the majority (93.4 %) were men. Approximately 77 % of patients lived in urban areas. Most patients (63 %) had their surgery in hospital A. As the total number of patients was less than ten in four hospitals for the reviewed time period, they have been collapsed into ‘Other’ category for the analysis. While smoking history was missing in 34 % of medical records, the proportion of current smokers was quite high in those with complete data (74 %). In the majority of patients, cancer was confined to bladder (56.6 %). About 86 % have had continent urinary diversion.

**Table 1:** Patient preoperative and operative characteristics

Characteristics^a^	All patients n = 273	No complications n = 194	In-hospital complications n = 79	*P* value
Age (years), mean (SD)	58.5 (8.9)	58.6 (8.5)	58.3 (9.8)	0.82
Age (years), n (%)				0.18
<55	97 (35.5)	66 (34.0)	31(39.2)	
55–64	106 (38.8)	82 (42.3)	24 (30.4)	
≥65	70 (25.6)	46 (23.7)	24 (30.4)	
Male, n (%)	255 (93.4)	179 (92.3)	76 (96.2)	0.29
Place of living, n (%)				0.70
Urban	210 (76.9)	148 (76.3)	62 (78.5)	
Rural	63 (23.1)	46 (23.7)	17 (21.5)	
Hospital, n (%)				<0.01
A	172 (63.0)	132 (68.0)	40 (50.6)	
B	48 (17.6)	25 (12.9)	23 (29.1)	
C	34 (12.5)	28 (14.4)	6 (7.6)	
Other^b^	19 (7.0)	9 (4.6)	10 (12.7)	
Year of surgery, n (%)				0.58
2011/12	57 (20.9)	37 (19.1)	20 (25.3)	
2009/10	86 (31.5)	60 (30.9)	26 (32.9)	
2007/08	81 (29.7)	61 (31.4)	20 (25.3)	
2005/06	49 (17.9)	36 (18.6)	13 (16.5)	
BMI (kg/m^2^), mean (SD)	26 (4.6)	25.9 (4.5)	26.1 (5.0)	0.75
Current smoker, n (%)	133 (73.9)	96 (75.0)	37 (71.2)	0.59
Diabetes, n (%)	14 (5.1)	9 (4.6)	5 (6.3)	0.56
Hypertension, n (%)	52 (19.0)	37 (19.1)	15 (19.0)	0.99
Preoperative hydronephrosis, n (%)	67 (24.5)	49 (25.3)	18 (22.8)	0.67
Preoperative chemotherapy, n (%)	22 (8.1)	14 (7.2)	8 (10.1)	0.42
Any prior surgery, n (%)	105 (38.5)	76 (39.2)	29 (36.7)	0.70
Renal diseases, n (%)	49 (17.9)	34 (17.5)	15 (19.0)	0.78
Gastrointestinal diseases, n (%)	55 (20.1)	41 (21.1)	14 (17.7)	0.52
IHD, n (%)	38 (13.9)	20 (10.3)	18 (22.8)	0.01
COPD/asthma, n (%)	20 (7.3)	14 (7.2)	6 (7.6)	0.91
Other diseases, n (%)	51 (18.7)	37 (19.1)	14 (17.7)	0.80
Hemoglobin level (g/l), mean (SD)	134.1 (18.7)	133.5 (19.2)	135.5 (17.5)	0.43
Preoperative red blood cells count (×10^12^/L), mean (SD)	4.2 (0.6)	4.1 (0.6)	4.2 (0.5)	0.36
Preoperative white blood cells count (×10^9^/L), mean (SD)	7.5 (2.1)	7.4 (1.9)	7.6 (2.6)	0.55
Preoperative creatinine level (mkm/L), mean (SD)	90.7 (24.8)	89.5 (24.6)	93.7 (25.2)	0.21
Preoperative glucose level (mmol/L), mean (SD)	5.7 (1.9)	5.8 (2.1)	5.2 (1.28)	0.01
Preoperative protein level (g/100 mL), mean (SD)	7.8 (0.6)	7.8 (0.59)	7.8 (0.58)	0.93
Preoperative fibrinogen (mg/100 mL), mean (SD)	430.2 (120.6)	430.9 (127.5)	428.4 (102.0)	0.87
Transurethral resection (TUR) in the past, n (%)	123 (45.1)	90 (46.4)	33 (41.8)	0.49
Preoperative cystoscopy, n (%)	76 (27.9)	48 (24.9)	28 (35.4)	0.08
Preoperative TUR biopsy, n (%)	60 (22.0)	39 (20.3)	21 (26.6)	0.26
Stage of the cancer, n (%)				0.57
Bladder confined (pT1-2 pN0 M0)	154 (56.6)	113 (58.5)	41 (51.9)	
Locally Advanced (pT3-4a pN0 M0)	86 (31.6)	59 (30.6)	27 (34.2)	
Extravesical (pT4b/any pT with N1-N3 or M1)	32 (11.8)	21 (10.9)	11 (13.9)	
Type of urinary diversion, n (%)				0.62
Continent	234 (85.7)	165 (85.1)	69 (87.3)	
Conduit	39 (14.3)	29 (14.9)	10 (12.7)	
ASA score, n (%)				0.01
1 or 2	180 (71.7)	135 (76.3)	45 (60.8)	
3 or 4	71 (28.3)	42 (23.7)	29 (39.2)	
Anesthesia type (%)				0.05
General	38 (13.9)	22 (11.3)	16 (20.3)	
Combined general and epidural	235 (86.1)	172 (88.7)	63 (79.7)	
Operative time (minutes), mean (SD)	325.6 (81.5)	322.6 (82.0)	333.0 (80.0)	0.34
Transfusion of RBC^c^, n (%)	80 (29.3)	53 (27.3)	27 (34.2)	0.26
Transfusion of FFP^c^, n (%)	55 (20.1)	21 (10.8)	34 (43.0)	<0.01
Any transfusion (RBC/FFP)^c^, n (%)	107 (39.2)	62 (32.0)	45 (57.0)	<0.01

*ASA* American Society of Anesthesiologists, *BMI* body mass index, *COPD* chronic obstructive pulmonary disease, *ICU* intensive care unit, *IHD* ischemic heart disease, *FFP* fresh frozen plasma, *LOS* length of stay, *RBC* red blood cells, *SD* standard deviation, *TUR* transurethral resection

^a^More than 5 % missing values present for the variables “Current smoker”(34 %), “BMI” (9 %), and “ASA” (8 %). For variables with missing values, calculations were made after excluding missing values

^b^Combined data from four hospitals

^c^Represents the number of patients with any intraoperative and/or postoperative transfusion

### Postoperative (in-hospital) complications

In total, 79 patients (28.9 %) developed at least one postoperative complication (Table 2). Among patients with complications, 54 (19.8 %) had one complication, 20 (7.3 %) had two and 5 (1.9 %) patients had three or more. The most commonly occurring complications were wound-related (n = 28 patients), followed by gastrointestinal (GI) (n = 25), and infectious complications (n = 19). Some complications were potentially life-threatening including sepsis observed in 0.7 %, myocardial infarction in 1.5 %, pulmonary embolism in 1.5 %, disseminated intravascular coagulation in 0.3 %, and stroke in 0.3 % of the sample. The in-hospital mortality rate was 4.8 % (n = 13) and all patients that died developed at least one complication before death.

**Table 2:** Postoperative in-hospital complications (n = 273)

Category: n (%)	Complication type	n (%)
Wound: n = 28 (10.3)	Wound infection	19 (7.0)
	Wound dehiscence	9 (3.3)
Gastrointestinal: n = 25 (9.2)	Postoperative ileus	20 (7.3)
	Anastomotic bowel leak	3 (1.1)
	Gastrointestinal bleeding	2 (0.7)
Infectious: n = 19 (7.0)	Urinary tract infection	13 (4.8)
	Postoperative sepsis	2 (0.7)
	Peritonitis	4 (1.5)
Cardiac: n = 11 (4.0)	Congestive heart failure	6 (2.2)
	Myocardial infarction	4 (1.5)
	Arrhythmia	1 (0.3)
Thromboembolic: n = 7 (2.6)	Deep venous thrombosis	3 (1.1)
	Pulmonary embolism	4 (1.5)
Pulmonary: n = 6 (2.1)	Postoperative pneumonia	6 (2.1)
Genitourinary: n = 4 (1.5)	Postoperative acute renal failure	3 (1.1)
	Urinary leak	1 (0.3)
Bleeding: n = 4 (1.5)	Postoperative bleed (not gastrointestinal)	3 (1.1)
	Disseminated intravascular coagulation	1 (0.3)
Miscellaneous: n = 3 (1.1)	Lymphocele	3 (1.1)
Neurological: n = 2 (0.7)	Ischemic stroke	1 (0.3)
	Peripheral neuropathy	1 (0.3)
Surgical: n = 2 (0.7)	Bowel perforation	2 (0.7)
Any complication: n = 79 (28.9)		

Compared to patients without complications, those who developed complications were more likely to have ischemic heart disease (IHD), lower blood glucose levels prior surgery, and higher anesthetic risk (Table 1). About 57 % of patients with complications had perioperative transfusion of red blood cells or fresh frozen plasma compared to 32 % in patients without complications. Among patients discharged alive, patients with complications had significantly longer length of preoperative, postoperative and total hospital stays, which is shown in Additional file 1: Figure S1. For example, the average total hospital LOS was 36 days (SD = 18) in the full sample, 49 days (SD = 20) in patients with complications and 32 days (SD = 15) in those without.

### Predictors of postoperative complications

We selected eight candidate variables for inclusion into the multivariable logistic regression model: age, gender, hospital, IHD, creatinine, glucose, American Society of Anesthesiologists score, and any perioperative transfusion of fresh frozen plasma or red blood cells (Table 3). The final model included four independent predictors (Table 3), and had acceptable calibration (Hosmer–Lemeshow goodness-of-fit test, *X*-squared = 5.9617 for *df* = 8, *P* value = 0.652) and discrimination (area under the ROC curve, C-statistic = 0.715). Based on adjusted analysis, IHD increased the odds of postoperative complications by more than three times (OR = 3.3, 95 % confidence interval (CI) 1.5–7.4), any postoperative transfusion increased the odds twice (OR = 2.0, 95 % CI 1.1–3.64), patients in hospital C had more than twice higher odds of complications compared to hospital A (OR = 2.3, 95 % CI 1.1–4.7), and higher preoperative glucose levels decreased the odds of complications (OR = 0.7, 95 % CI 0.6–1.0).

**Table 3:** Univariable and multivariable analyses of predictors of postoperative complications

Variables	Univariable analysis		Multivariable analysis	
	OR (95 % CI)	*P* value	OR (95 % CI)	*P* value
Age (years)				
<55 *(reference)*	1.00		–	
55–64	0.62 (0.33–1.16)	0.137		
≥65	1.11 (0.58–2.13)	0.752		
Gender				
Female *(reference)*	1.00		–	
Male	2.12 (0.68–9.36)	0.245		
Hospital				
A *(reference)*				
B	3.67 (1.39–9.80)	0.009	2.05 (0.67–6.23)	0.20
C	3.04 (1.56–5.94)	0.001	2.25 (1.07–4.70)	0.03
Other	0.71 (0.25–1.73)	0.475	0.83 (0.28–2.11)	0.73
IHD	2.57 (1.27–5.18)	0.008	3.31 (1.48–7.44)	<0.01
Creatinine (mkm/L)	1.00 (0.99–1.02)	0.215	–	
Glucose (mmol/L)	0.76 (0.60–0.93)	0.014	0.71 (0.63–0.95)	0.02
ASA score				
1 or 2 *(reference)*			–	
3 or 4	2.074 (1.15–3.70)	0.014		
Any transfusion (RBC/FFP)	2.82 (1.65–4.85)	<0.001	1.99 (1.10–3.64)	0.02

*ASA* American Society of Anesthesiologists, *CI* confidence interval, *FFP* fresh frozen plasma, *IHD* ischemic heart disease, *OR* odds ratio, *RBC* red blood cells

## Discussion

The study established the surgical volume and incidence of in-hospital complications of RC for bladder cancer in Armenia from 2005 to 2012, and assessed the predictors of these complications. To our knowledge this was the first study conducted in Armenia among patients with RC, with reasonable generalizability to the entire population of patients in Armenia for the specified time period. We found that 273 patients had RC in eight Armenian hospitals, and the incidence of postoperative complications and mortality was quite substantial.

Except age, the baseline profile of Armenian patients was not very different from those in other countries (Konety et al. 2006; Stimson et al. 2010; Kim et al. 2012; van Hemelrijck et al. 2013; Aziz et al. 2014). At the time of surgery Armenian patients were 58.5 years old on average. The average age of 679 RC patients in 18 European centers in 2011, for example, was 70 years (Aziz et al. 2014), and the average age of 50,625 RC patients in the US Nationwide Inpatient Sample from 2001 to 2008 was 68.4 years (Kim et al. 2012). This indicates that bladder cancer may affect Armenian patients at younger ages. Armenian men also get exposed to smoking, the major risk factor of bladder cancer, at quite a young age. In 2001, the average age of smoking initiation was 18.5 in Armenia men and 28.0 in women (Gilmore et al. 2004). In our study, about 74 % of patients reported being current smokers, and smoking continues to be a major public health problem in Armenia.

The observed in-hospital mortality rate in our sample was 4.8 %, somewhat higher than the mortality rates reported in other studies (Konety et al. 2006; Shabsigh et al. 2009; Kim et al. 2012; Maffezzini et al. 2008). However, it is hard to conclude if this is Han indication of a sicker Armenian surgical population or inadequate pre- or postoperative management. Furthermore, the proportion of patients developing at least one postoperative complication was 29 %, well within the range of reported complication rates in other studies that varied from 21.8 to 57 % (Konety et al. 2006).

The most common complications observed in our study were postoperative ileus (7.3 %), followed by wound infections (7.0 %), urinary tract infections (4.8 %), and wound dehiscence (3.3 %). The study in 2538 RC patients by Hollenbeck et al. similarly reported the top four most common complications as ileus (9.7 %), urinary tract infections (7.8 %), wound dehiscence (5.5 %), and wound infections (5.2 %) (Hollenbeck et al. 2005), though this study included a relatively older patient population (65.5 years old) (Hollenbeck et al. 2005). Using a reporting methodology by Shabsigh et al. (Shabsigh et al. 2009), we found that wound complications had the highest incidence in our study population affecting 10 % of patients, followed by GI complications (9 %), and infectious complications (7 %). Shabsigh et al. also reported that these three complication categories were the most frequently occurring in a hospital in New York; however, GI complications were observed in 29 %, infectious in 25 % and wound complications in 15 % of patients (Shabsigh et al. 2009). In the study by Takada et al. among Japanese RC patients the highest incidence was observed for infectious complications (30 %), followed by GI (26 %) and wound (21 %) complications (Takada et al. 2012). These two studies, however, captured 90-day complications (Shabsigh et al. 2009; Takada et al. 2012) while we recorded only in-hospital complications.

Similar to other past studies, we found that postoperative complications significantly increase the hospital LOS of patients (Shabsigh et al. 2009; Konety and Allareddy 2007). Shabsigh et al. reported that LOS is increasing with the increase of the severity of complications (Shabsigh et al. 2009). Another study further reported that the addition of each complication significantly increased the risk of in-hospital mortality, LOS and costs (Konety and Allareddy 2007). The total hospital LOS in our patient sample was higher than in other studies, which could be explained by differences in healthcare systems (Shabsigh et al. 2009; Konety and Allareddy 2007; Maffezzini et al. 2008). Since all centers that conduct RC in Armenia are located in the capital city, patients from remote areas stay longer until they recover and are fit to travel. Furthermore, in Armenia medical costs are mostly covered by patients and the major cost driver is the cost of the surgery and not the length of hospital stay after the procedure.

Not all risk factors identified by our literature review have been proven to be predictive of RC complications in Armenia. For example, several studies reported an increased risk of postoperative complications with increasing age (Hollenbeck et al. 2005; Konety et al. 2006; Kim et al. 2012; Gore et al. 2010). This was not true for the Armenian population, most probably because of a relatively younger patient population. The risk of RC complications by gender has been controversial in the literature with some studies reporting an increased risk for females (Shabsigh et al. 2009; Gore et al. 2010), increased risk for males (Kim et al. 2012; Takada et al. 2012), or no risk difference (Konety et al. 2006; Lee et al. 2004) as in our sample.

The evidence is controversial regarding the issue of surgical volume and patient outcomes. While some studies supported the positive impact of higher surgical caseload and hospital volume on RC outcomes (Kim et al. 2012; McCabe et al. 2007), others showed no difference (Konety et al. 2006; Takada et al. 2012; Gore et al. 2010). There is no unified approach to define high-, medium- or low-volume hospital categories for RC. The study by Kim et al. in 1173 US hospitals found that patients in high volume hospitals (defined as ≥5RC/year) had 33 % less chance of in-hospital complications and 40 % less chance of in-hospital mortality than patients treated in low volume hospitals (defined as <1.5 RC/year) (Kim et al. 2012). Another study that defined >10 RC/year as high, 5–10 as medium and ≤5 as low-volume hospitals, found no association between volume and risk of complications (Takada et al. 2012). A recent US study reported that surgeons who perform ≥7 RC per year had 44 % lower odds of major complications at 90 days than those performing 1 RC per year (Leow et al. 2015). We, however, did not capture per surgeon surgical volume. In our sample, the majority of patients underwent surgery in one of the hospitals, hospital A, where the RC volume was on average 22/year. The RC volume was 6, 4 and <1 per year in hospital B, C, and in ‘other’ category. The odds of complications were almost twice higher in patients in hospital C than in hospital A.

ASA scores above 2 have been shown to increase the risk of RC complications (Hollenbeck et al. 2005; Novotny et al. 2011; Shabsigh et al. 2009; Takada et al. 2012). In our study, ASA score was not an independent predictor of complications. The comorbidities that have been shown to increase the risk of complications are also variable between published studies (Hollenbeck et al. 2005; Novotny et al. 2011; Kim et al. 2012; Takada et al. 2012; Gore et al. 2010). Such differences can be explained by differences in the prevalence in the index population, in over- or under-reporting, and in peri-operative management. In our sample, for example, although about 74 % of patients (with complete information) reported current smoking history, the recorded prevalence of COPD was only 7 %. In our study IHD was one of the strongest predictors, increasing the odds of developing in-hospital complications by three times. Most past studies either did not capture IHD as a separate patient baseline factor (Konety et al. 2006; Kim et al. 2012), or captured other cardiovascular disease entities (Hollenbeck et al. 2005; Gore et al. 2010). Considering our finding, we think that future studies should report the prevalence of IHD in this population and further investigate its effect on outcomes.

With detailed review of medical records we captured several pre-operative factors that are not available through administrative databases. In bivariate analysis, we found that patients without complications had significantly higher glucose levels than those who developed in-hospital complications. In multivariable analysis, higher glucose levels were associated with lower odds of complications. Our review of past studies did not find any study in RC patient population that evaluated baseline glucose levels and their effect on surgical outcomes. Considering the retrospective nature of the study, it is impossible to conclude if the low glucose levels are a result of advance cancer stages and derangements in carbohydrate metabolism. We were also unable to fully investigate the perioperative management of patients with low or high glucose levels.

Based on our findings, any intraoperative and/or postoperative transfusion of fresh frozen plasma or red blood cells was another independent predictor, almost doubling the odds of post-operative complications. Intraoperative blood transfusion was a significant predictor of in-hospital complications in a study of 2538 RC patients by Hollenbeck et al. (OR = 1.4, 95 % CI 1.2–1.7) (Hollenbeck et al. 2005). While several past studies did not report patient blood loss or transfusion of blood products (most likely because of the use of administrative databases that generally lack such data), transfusion of blood products has been shown to increase the risk of adverse surgical outcomes in cardiac surgery (Horvath et al. 2013), knee arthroplasty (Hart et al. 2014), and neurosurgery (Rolston et al. 2014).

Potential limitations of our study warrant discussion. There is always some degree of variability in medical records and data capture methods between different hospitals. To minimize this, we used a standardized data collection tool. Smoking history, which was a variable of high interest, was not recorded in almost one third of medical records, and had to be excluded from adjusted analysis. As the information was collected retrospectively, it was impossible to accurately evaluate the amount of blood loss, the appropriateness of indications for transfusions, and reasons and management of low glucose levels. At the same time, one of the major strengths of our study was the use of detailed clinical information from medical records that was somewhat limited in other studies that used administrative databases (Hollenbeck et al. 2005; Konety et al. 2006; Kim et al. 2012; Gore et al. 2010). This allowed us to generate new hypotheses regarding the role of glucose levels and IHD in RC complications. We also used a relatively recent cohort of patients, and obtained a national sample of RC patients. The results of our study can be used for comparisons with studies from other low or middle income countries.

## Conclusion

The incidence of postoperative complications of RC in Armenia was similar to those observed in other countries. Coronary artery disease, receiving transfusion, glucose levels and hospital type were found to be independent predictors of complications. Future prospective studies should evaluate the effect of RC complications on long-term outcomes and costs in Armenia. National guidelines should address the appropriateness of perioperative transfusion, and propose evidence-based management approaches for IHD patients. Surgical care pathways should be developed to standardize patient care in all centers. Policy recommendations should address surgeon training and hospital volume requirements to decrease the risk of RC complications.

## Methods

The study utilized a retrospective chart review in a cohort of patients who had RC followed by either continent or conduit urinary diversion in Armenia from January 1, 2005 to December 31, 2012, following them from the day of index hospital admission for RC to the day of discharge or in-hospital death, whichever occurred first. We excluded patients with ureterocutaneostomy because these are mostly patients with reduced life expectancy (more advanced cancer stages) or multiple comorbidities with high risk of non-diversion related early postoperative complications (Pycha et al. 2008). We considered only cases with continent or conduit urinary diversion to have a more homogenous patient population in terms of postoperative complications related to bowel involvement.

To identify patients, first we selected all hospitals in the capital city of Armenia, Yerevan that had urological departments and had a potential capacity to perform RC. Then we contacted the heads of the departments to establish if this surgery was performed in their hospitals and to inquire if they know of any other centers performing RC. The study timeframe was limited to 7 years to minimize the potential biases due to changes in clinical practice, care documentation, or healthcare regulations on observed outcomes.

### Ethical approval

All procedures performed in studies involving human participants were in accordance with the ethical standards of the institutional research committee and with the 1964 Helsinki declaration and its later amendments or comparable ethical standards. The study protocol was approved by the Institutional Review Board of the American University of Armenia. For this type of study (retrospective study) formal individual patient consent was not required.

### Data collection

We conducted a detailed medical chart abstraction of all patients in the sample. A previously described framework for assessing postoperative complications after RC was used as a starting point to define data abstraction elements (Hollenbeck et al. 2005). The framework categorizes data elements as *preoperative risk factors* (e.g., demographic characteristics, comorbidities, cancer staging), *structure of care factors* (e.g., year, hospital), *process of care factors* (e.g., perioperative transfusion, operative time), and *outcomes* (i.e., complications, mortality, length of stay). The final data dictionary was developed after a review of peer-reviewed literature and consulting content experts. Postoperative complications were categorized following a standardized reporting methodology suggested by Shabsigh et al. (2009). One of the authors, a practicing urologist, pre-tested the data collection tool in 10 patient records that were not part of the study. Then, for consistency in abstracting medical information, the same researcher used the data collection tool in all participating hospitals.

### Statistical analysis

All statistical analyses were performed using R software (R Core Team 2014 Foundation for Statistical Computing, Vienna, Austria). Continuous variables were described using means and standard deviations, and categorical variables were described using frequencies and percentages. Independent t test or Wilcoxon test was used to compare continuous variables, and Chi square test to compare categorical variables. Loess smooth curves were used to investigate the relationship between the continuous variables and the outcome (i.e., postoperative complications). Multivariable logistic regression analysis was applied to estimate the independent risk factors of developing “any postoperative complication”. Candidate variables for the model were specified a priori, including predictors identified from the literature and those that were deemed clinically meaningful by content experts. Variables with more than 10 % missing values were not considered for the regression analysis. All candidate variables were first included into the model and then removed one by one using the log-likelihood ratio test and Akaike information criterion. Variables were tested for potential confounding, interactions and multicollinearity. Model fit was tested using diagnostic plots, the Hosmer–Lemeshow goodness-of-fit test, and area under the receiver operating characteristic (ROC) curve (Hosmer et al. 2013). All results with the P value less than 0.05 were considered statistically significant.

## Additional file

10.1186/s40064-016-1757-9 Distribution of length of stay (LOS) variables.
