# Supplementary figures and images for: Risk factors of postoperative complications after radical cystectomy with continent or conduit urinary diversion in Armenia
Source: Springerplus. 2016 Feb 20;5:134. doi: 10.1186/s40064-016-1757-9 (PMC4761360; doi:10.1186/s40064-016-1757-9)

Figure S1.
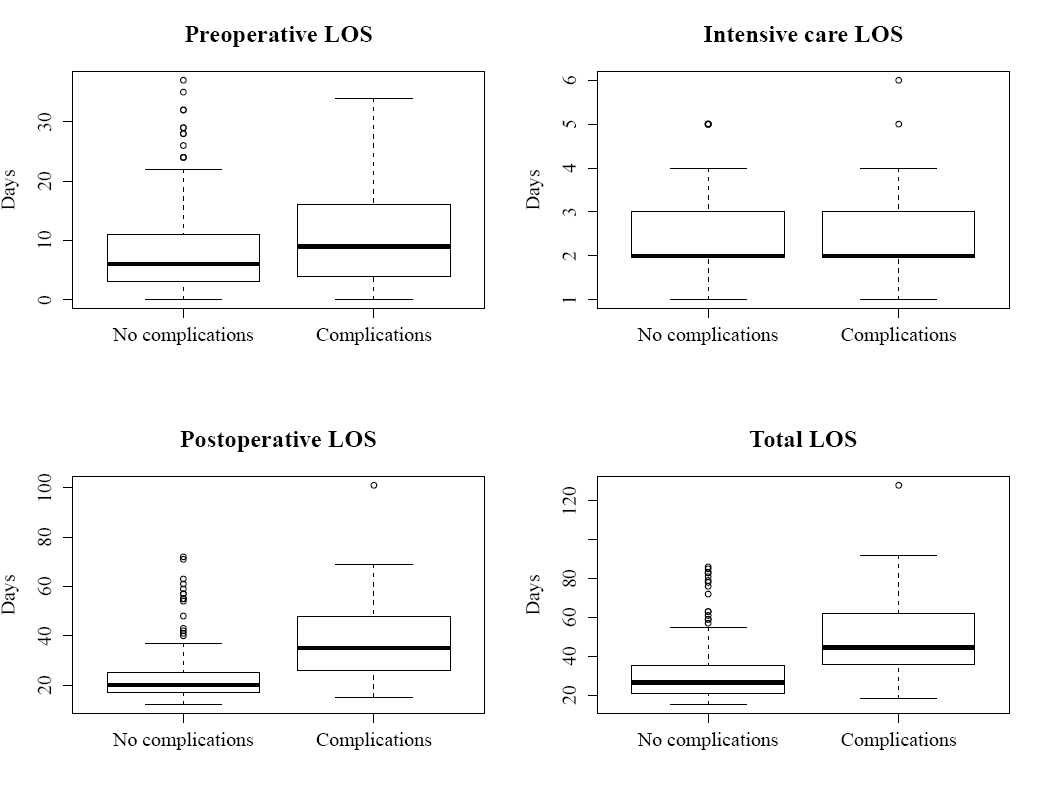

Supplement: Supplementary file 1 — 10.1186/s40064-016-1757-9 Distribution of length of stay (LOS) variables. [file 40064_2016_1757_MOESM1_ESM.docx]
